# Epidemiology and predictors of traumatic spine injury in severely injured patients: implications for emergency procedures

**DOI:** 10.1007/s00068-020-01515-w

**Published:** 2020-10-06

**Authors:** David Häske, Rolf Lefering, Jan-Philipp Stock, Michael Kreinest, The TraumaRegister DGU

**Affiliations:** 1 German Red Cross, Emergency Medical Service, Obere Wässere 1, 72764 Reutlingen, Germany; 2 Center for Public Health and Health Services Research, University Hospital Tübingen, Tübingen, Germany; 3 Institute for Research in Operative Medicine (IFOM), University Witten/Herdecke, Cologne, Germany; 4 Department of Anesthesiology, Intensive Care Medicine, Emergency and Pain Medicine, Klinikum am Steinenberg, Reutlingen, Germany; 5 Department of Trauma and Orthopedic Surgery, BG Trauma Center Ludwigshafen, Ludwigshafen, Germany; 6 Committee on Emergency Medicine, Intensive Care and Trauma Management (Sektion NIS) of the German Trauma Society (DGU), Cologne, Germany

**Keywords:** Immobilization, Prehospital, Risk, Trauma

## Abstract

**Purpose:**

This study aimed to identify the prevalence and predictors of spinal injuries that are suitable for immobilization.

**Methods:**

Retrospective cohort study drawing from the multi-center database of the TraumaRegister DGU^®^, spinal injury patients ≥ 16 years of age who scored ≥ 3 on the Abbreviated Injury Scale (AIS) between 2009 and 2016 were enrolled.

**Results:**

The mean age of the 145,833 patients enrolled was 52.7 ± 21.1 years. The hospital mortality rate was 13.9%, and the mean injury severity score (ISS) was 21.8 ± 11.8. Seventy percent of patients had no spine injury, 25.9% scored 2–3 on the AIS, and 4.1% scored 4–6 on the AIS. Among patients with isolated traumatic brain injury (TBI), 26.8% had spinal injuries with an AIS score of 4–6. Among patients with multi-system trauma and TBI, 44.7% had spinal injuries that scored 4–6 on the AIS. Regression analysis predicted a serious spine injury (SI; AIS 3–6) with a prevalence of 10.6% and cervical spine injury (CSI; AIS 3–6) with a prevalence of 5.1%. Blunt trauma was a predictor for SI and CSI (OR 4.066 and OR 3.640, respectively; both *p* < 0.001) and fall > 3 m for SI (OR 2.243; *p* < 0.001) but not CSI (OR 0.636; *p* < 0.001). Pre-hospital shock was predictive for SI and CSI (OR 1.87 and OR 2.342, respectively; both *p* < 0.001), and diminished or absent motor response was also predictive for SI (OR 3.171) and CSI (OR 7.462; both *p* < 0.001). Patients over 65 years of age were more frequently affected by CSI.

**Conclusions:**

In addition to the clinical symptoms of pain, we identify ‘4S’ [spill (fall) > 3 m, seniority (age > 65 years), seriously injured, skull/traumatic brain injury] as an indication for increased attention for CSIs or indication for spinal motion restriction.

## Introduction

According to the literature, only 1–2% of all trauma patients suffer from relevant spinal injuries [1]. These patients are at risk of spinal cord injuries (SCIs) with severe neurological consequences, which occur in approximately 20% of these cases [2]. Because spinal immobilization can reduce spinal movement [3], the immobilization of patients with SCIs has been recommended since the mid-twentieth century to prevent further damage. Since then, immobilization has become a standard procedure in emergency medicine [2]; however, a recent study found that 20% of patients suffering from a cervical SCI were not immobilized whatsoever by emergency care providers [4]. A possible explanation is the fact that, over the years, no evidence for the benefit of immobilization for patient outcomes has been obtained from rigorous studies (e.g., randomized controlled trials) [5–7]. Furthermore, regardless of the method, immobilization has its disadvantages, such as general patient manipulation, pain, decubitus, and prolonged pre-hospital time [8, 9].

Therefore, a differentiated indication is recommended. In many cases, decision rules are established based on the circumstances of the accident (e.g., accident mechanism) and the patient’s symptoms, but also from studies with healthy volunteers or corpses, whose results were generalized to trauma patients [5, 7]. The most well-known decision-making aids for spinal immobilization are the NEXUS criteria and the Canadian C-Spine Rule (CCSR), and, in Europe, the E.M.S. IMMO protocol and the Scandinavian recommendations [10–13]. All of these decision rules include possible predictors that should detect an increased probability of spinal injury in trauma patients.

However, the decision rules were often created for the indication of imaging to establish a definitive diagnosis; they only partially evaluated for pre-hospital care [12–14]. But, for emergency procedures, however, the aim is not to make an exact diagnosis but to identify the indication for immobilization to prevent possible consequential damage during extrication and transport.

From this point of view, studies on predictors must be considered.

In an 8-year European cohort study of patients with spinal fractures, Hasler et al. showed that falls from > 3 m were the strongest predictor for spine trauma [15]. In addition, cervical spine injuries were most common in traffic accidents, sports, and falls > 2 m, as well as in patients > 65 years-old [16]. A registry study on spinal cord injuries showed that traumatic brain injuries (TBIs), shock at the scene, the severity of injuries, and age > 60 years, worsen the outcome [17]. While anatomy has not changed over the decades, leisure behaviors and safety techniques have (e.g., wearing seatbelts in vehicles) [18, 19], and with it the question of the generalizability of non-European studies [12, 13, 20].

### Goals of this investigation

As such, this study aims to identify the epidemiological characteristics and possible predictors of spinal injury in severely injured patients in Europe.

## Materials and methods

### Study design

Data from the TraumaRegister DGU^®^ (TR-DGU) were retrospectively analyzed.

### Database

The TR-DGU of the German Trauma Society [Deutsche Gesellschaft für Unfallchirurgie (DGU)] was founded in 1993. The aim of this multi-center database is to collect pseudonymized and standardized documentation of severely injured patients. Data are collected prospectively in four consecutive phases from the site of the accident until discharge from hospital: pre-hospital phase, emergency room and initial surgery phase, intensive care phase, and discharge phase. The documentation includes detailed information on demographics, injury pattern, comorbidities, pre- and in-hospital management, the course in the intensive care unit (ICU), relevant laboratory findings (including data on transfusion), and the outcomes of each patient. The inclusion criteria comprised (1) hospital admission via an emergency room with subsequent ICU care; (2) vital signs upon hospital arrival; or (3) death prior to ICU admission.

The infrastructure for documentation, data management, and data analysis is provided by the Academy for Trauma Surgery (AUC; Akademie der Unfallchirurgie GmbH), a company affiliated to the German Trauma Society. The scientific leadership is provided by the Committee on Emergency Medicine, Intensive Care and Trauma Management (Sektion NIS) of the German Trauma Society. The participating hospitals submit their pseudonymized data to a central database via a web-based application. The participating hospitals are primarily located in Germany (90%); however, an increasing number of foreign hospitals also contribute data, including hospitals from Austria, Belgium, China, Finland, Luxembourg, Slovenia, Switzerland, The Netherlands, and the United Arab Emirates. Currently, approximately 33,000 cases from over 650 hospitals are entered into the database per year. Participation in TR-DGU is voluntary. For hospitals associated with TraumaNetzwerk DGU^®^, however, the entry of at least a basic data set is obligatory for reasons of quality assurance. The present study is in line with the publication guidelines of the TR-DGU, underwent an internal peer review and is registered as TR-DGU project ID 2019-028.

### Classifications

#### Injury severity assessment

The Abbreviated Injury Scale (AIS) is an anatomical coding system for classifying and describing the severity of injuries in every body region, and it is used in many trauma registries. Based on the AIS values, the injury severity score (ISS) can be calculated to assess the cumulative trauma severity. Seriously injured is defined by an ISS ≥ 16.

#### Motor function assessment

Neurological assessment was performed according to the Eppendorf–Cologne Scale (ECS) [21]. The ECS is a prognostic score for TBI that is based on the motor component and pupil response and size specified in the Glasgow Coma Scale (GCS). In our analysis, we exclusively used the motor response, which is classified as normal (0 points), specific (1 point), nonspecific (2 points), or completely absent (3 points). According to the literature, the ECS shows a significantly higher accuracy for the prediction of TBI presence compared to the GCS [22].

### Patient selection

**Fig. 1:**
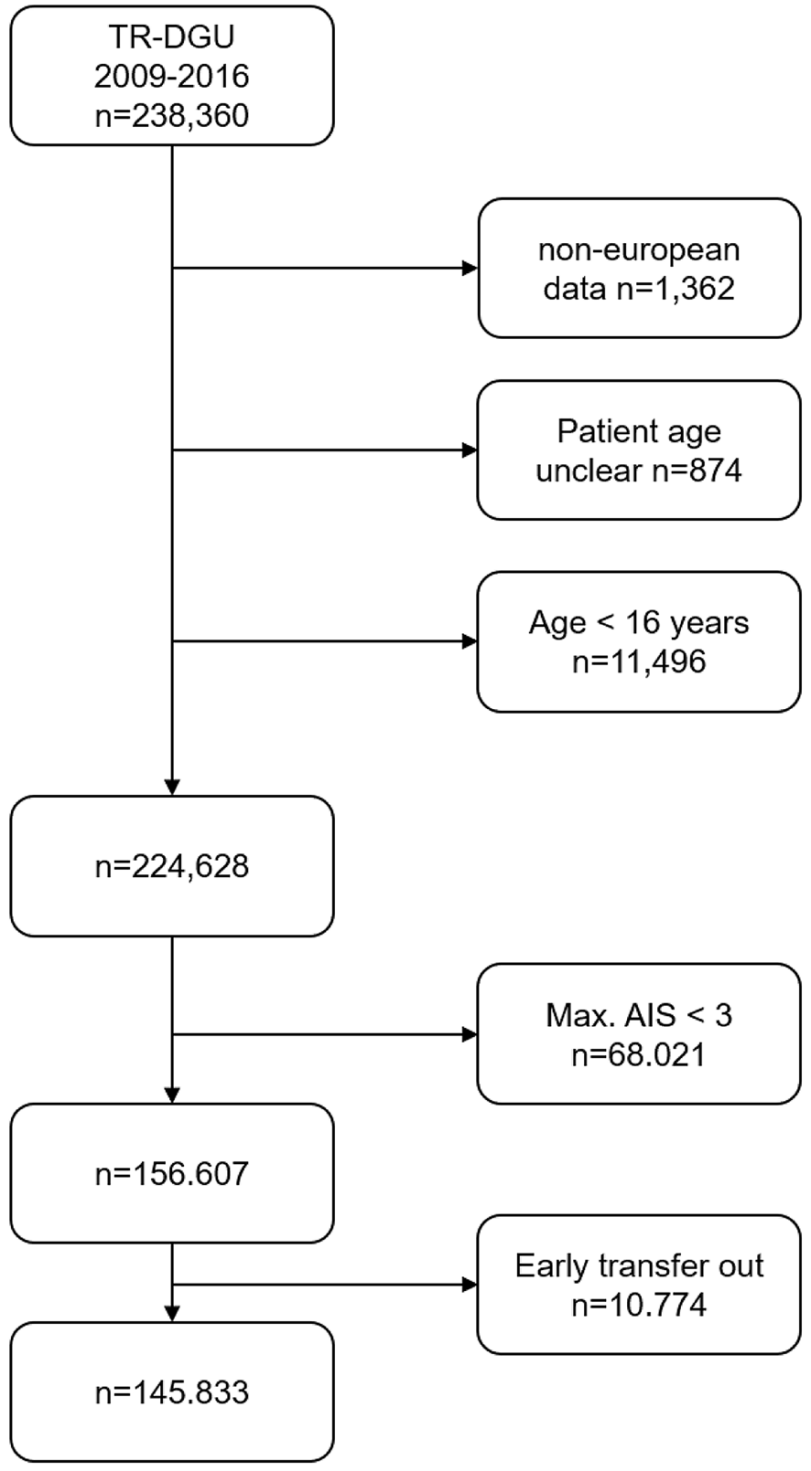
Flowchart representing the inclusion and exclusion criteria

Patients aged 16 years or older who were treated primarily in a participating hospital between 2009 and 2016 were enrolled. Further inclusion and exclusion criteria are summarized in Fig. 1.

### Epidemiological analysis

For the descriptive analysis of epidemiological data, we categorized spine injuries into three groups: none, AIS severity score of 2–3 (injuries with no or transient neurological sings), and AIS score of 4–6 (injuries with relevant neurological abnormalities). AIS scores of 1 were excluded because they are irrelevant to therapy or outcome in terms of severe injury care. For the regression analysis, an AIS 3–6 was chosen as a dependent variable because spine injuries classified as AIS ≥ 3 are characterized as relevant injuries [23]. This is different to the descriptive presentation of the data. But for the predictors, the clinical relevance was important regarding the relevant spine injuries and thus immobilization, which will be necessary already for spinal fractures with an elevated risk of instability but without relevant neurological abnormalities, as well as for injuries of the spinal cord and other neural structures with neurological symptoms (AIS 3–6). AIS 2 injuries are often discovered later, are less relevant, and do not result in therapeutic decisions.

### Analysis of potential predictors

To identify potential predictors that can predict severe spinal injuries (i.e., AIS ≥ 3), a linear regression was performed with age, accident mechanism, accompanying injuries, vital signs, and motor response as potential predictors according to the ECS.

### Statistical analysis

Data are presented as mean with standard deviation (± SD) for continuous variables and as number of cases with percentages for categorical variables. *p* values < 0.05 were considered statistically significant. For the descriptive patient data, no *p* values were given. Logistic regression was performed to identify predictors with a relevant impact on the main question. Regression coefficients are given with standard error and the respective *p* value of the model, as well as odds ratio (OR) with 95% confidence interval (CI). Statistical analysis was performed using SPSS v.24.0 (IBM Inc., Armonk, NY, USA).

## Results

During the study period from 2009 to 2016, 145,833 patients met the inclusion criteria (Fig. 1). The mean age was 52.7 ± 21.1 years, and 70.7% of the patients were male. The hospital mortality rate was 13.9% (*n* = 20,234), and the mean ISS was 21.8 ± 11.8.

### Epidemiological analysis

Regarding the severity of spinal injuries, a total of 102,152 patients (70.0%) had no spine injury, 37,749 patients (25.9%) were classified as AIS 2–3, and 5932 patients (4.1%) were classified as AIS 4–6 (Table 1). Among patients with spine injuries, cervical injuries were sustained by 11,095 (25.4%) patients, the thoracic spine was injured in 17,517 (40.1%) patients, and the lumbar spine was injured in 18,974 (43.4%) patients (multiple choice). Spine injuries with AIS 4–6 were most common in patients between 60 and 79 years of age (Table 1); however, patients over the age of 80 years often sustained only minor spinal injuries. Thoracic and abdominal injuries were most prevalent in the AIS 2–3 group, while accompanying injuries to the head, face, and extremities were found mostly without injuries to the spine. Patients with TBI had an AIS 4–6 spinal injury score in 26.8% of cases. Spine injures was even more evident in patients with TBI who had combined multi-system trauma; here, 44.7% had spinal injuries with an AIS score of 4–6.

**Table 1:** Demographic, injury, and vital parameter data of patients

	No spinal injury	AIS 2–3	AIS 4–6	Overall patients
Age 16–59 years 60–69 years 70–79 years ≥ 80 years	*n* (%) 60,580 (59.3%) 12,611 (12.3%) 15,193 (14.9%) 13,768 (13.5%)	*n* (%) 24,260 (64.3%) 4952 (13.1%) 5078 (13.5%) 3459 (9.2%)	*n* (%) 3701 (62.4%) 812 (13.7%) 886 (14.9%) 533 (9.0%)	*n* (%) 88,541 (60.7%) 18,375 (12.6%) 21,157 (14.5%) 17,760 (12.2%)
Relevant injuries (AIS ≥ 3) Head Face Thorax Abdomen Extremities Isolated TBI Multisystem trauma with TBI	*n* (%) 47,210 (46.2%) 5105 (5.0%) 42,180 (41.3%) 9172 (9.0%) 24,740 (24.2%) 21,965 (21.5%) 33,119 (21.5%9	*n* (%) 12,584 (33.3%) 1756 (4.7%) 22,814 (60.4%) 4144 (11.0%) 7986 (21.2%) 1383 (3.7%) 20,073 (3.7%)	*n* (%) 1228 (20.7%) 135 (2.3%) 2383 (40.2%) 335 (5.6%) 545 (9.2%) 1587 (26.8%) 2653 (44.7%)	*n* (%) 61,022 (41.8%) 6996 (4.8%) 67,377 (46.2%) 13,651 (9.4%) 33,271 (22.8%) 24,935 (17.1%) 55,845 (38.3%)
Vital signs GCS prehospital GCS pre-hospital, median SBP prehospital Heart frequency prehospital SBP trauma room Heart frequency trauma room	Mean (SD) 12.21 (4.10) 14.00 133.94 (31.31) 90.42 (21.63) 133.27 (29.75) 87.22 (20.23)	Mean (SD) 12.55 (3.93) 15.00 128.40 (30.29) 92.71 (21.11) 127.92 (29.51) 90.12 (20.64)	Mean (SD) 12.36 (3.99) 14.00 119.36 (29.83) 84.82 (22.54) 121.58 (28.59) 83.53 (22.16)	Mean (SD) 12.31 (4.06) 14.00 131.96 (31.18) 90.83 (21.19) 131.44 (22.80) 87.89 (20.49)
ECS motor response Normal Specific Unspecific No response	*n* (%) 59,083 (64.9%) 16,643 (18.3%) 3660 (4.0%) 11,649 (12.8%)	*n* (%) 23,635 (69.1%) 5519 (16.1%) 1072 (3.1%) 3981 (11.6%)	*n* (%) 3086 (60.3%) 686 (13.4%) 120 (2.3%) 1229 (24.0%)	*n* (%) 85,804 (65.8%) 22,848 (17.5%) 4852 (3.7%) 16,859 (12.9%)
Minutes of patient care On-scene time (*n* = 38,955) Emergency department (*n* = 53,018)	Mean (SD) 28.4 (50.9) 69.4 (16.4)	Mean (SD) 29.9 (16.8) 72.3 (50.5)	Mean (SD) 30.15 (16.3) 78.17 (54.6)	Mean (SD) 28.9 (16.5) 70.6 (51.1)
Hospital SBP ≤ 90 mmHg Transfusion of packed red blood cells	*n* (%) 8042 (8.7%) 3007 (38.8%)	*n* (%) 4139 (11.9%) 1935 (47.4%)	*n* (%) 895 (16.9%) 306 (34.9%)	*n* (%) 13,076 (9.9%) 5248 (41.3%)
Diagnostic accuracy of pre-hospital emergency doctors Spine injury, none Spine injury, slight Spine injury, moderate Spine injury, serious	*n* (%) 20,247 (72.9%) 3068 (11.0%) 3259 (11.7%) 1213 (4.4%)	*n* (%) 4523 (35.4%) 1587 (12.4%) 4121 (32.2%) 2557 (20.0%)	*n* (%) 332 (13.2%) 84 (3.4%) 366 (14.6%) 1724 (68.8%)	*n* (%) 25,102 (58.3%) 4739 (11.0%) 7746 (18.0%) 5494 (12.8%)

*GCS* Glasgow Coma Scale, *SBP* systolic blood pressure, *TBI* traumatic brain injury, *ECS* Eppendorf–Cologne Scale

The vital signs, which are described in Table 1, show that the prehospital and trauma room mean blood pressure values were lowest in the AIS 4–6 group, whereas GCS and heart rate were highest in the AIS 2–3 group. The accuracy of the correct emergency-physician diagnosis of the spinal trauma was dependent on the severity of the injury. For this purpose, the severity of injury is subjectively assessed prehospital in categories that are compared to the final discharge diagnosis. While patients without spine injuries were overestimated in 27.1% of cases, patients with AIS 4–6 spinal injuries were underestimated in 31.2%. The most considerable variation was found in the AIS 2–3 group, where 47.8% of the patients were underestimated and 20% were overestimated.

### Analysis of potential predictors

Multivariate logistic regression analysis considered spine injury (AIS 3–6) with a prevalence of 10.6% and cervical spine injury (AIS 3–6) with a prevalence of 5.1% as dependent variables. Blunt trauma was a remarkable independent variable for spinal trauma (OR 4.066, *p* < 0.001) and cervical spine injury (OR 3.640, *p* < 0.001). A fall from over 3 m was a predictor for a spine injury (OR 2.243, *p* < 0.001), but not for cervical spine injury (0.636, *p* < 0.001). A pre-hospital GCS ≤ 8 did not suggest a spinal injury per se, but a pre-hospital systolic blood pressure ≤ 90 mmHg was predictive (spine OR 1.877, *p* < 0.001, and cervical spine OR 2.342, *p* < 0.001). Patients aged 65 years and older were more likely to have cervical spine injuries. The diminished or absent motor response was a likely injury for both the spine and cervical spine (Table 2).

**Table 2:** Multivariate logistic regression analysis to predict any relevant spine injury (AIS 3–6, *n* = 15,481; prevalence = 10.6%), and relevant cervical spine injury (AIS 3–6, *n* = 5929; prevalence = 5.1%)

Predictor	Complete spine				Cervical spine			
	Coefficient (SE)	OR	95% CI	*p* value	Coefficient (SE)	OR	95% CI	*p* value
Head AIS ≥ 3	− 1.655 (0.032)	0.191	0.180–0.203	< 0.001	− 1.700 (0.047)	0.183	0.166–0.200	< 0.001
Face AIS ≥ 3	− 0.307 (0.039)	0.736	0.681–0.795	< 0.001	− 0.133 (0.056)	0.876	0.784–0.978	= 0.018
Thorax AIS ≥ 3	− 0.996 (0.024)	0.369	0.352–0.387	< 0.001	− 1.418 (0.040)	0.242	0.224–0.262	< 0.001
Abdomen AIS ≥ 3	− 0.838 (0.043)	0.432	0.397–0.471	< 0.001	− 1.131 (0.077)	0.323	0.277–0.375	< 0.001
Extremities and pelvis AIS ≥ 3	− 1.421 (0.032)	0.241	0.227–0.257	< 0.001	− 1.679 (0.056)	0.186	0.167–0.208	< 0.001
Male	0.009 (0.025)	1.009	0.961–1.059	= 0.730	0.210 (0.039)	1.234	1.144–1.332	< 0.001
Blunt trauma	1.403 (0.092)	4.066	3.395–4.870	< 0.001	1.292 (0.143)	3.640	2.748–4.821	< 0.001
Accident mechanism (reference car)								
Motorcycle Bicycle Pedestrian Fall > 3 m Fall < 3 m Other	− 0.007 (0.038) − 0.138 (0.048) − 0.534 (0.061) 0.808 (0.031) − 0.050 (0.037) − 0.152 (0.045)	0.993 0.871 0.586 2.243 0.951 0.859	0.922–1.070 0.793–0.956 0.520–0.660 2.109–2.385 0.885–1.022 0.787–0.939	= 0.852 = 0.004 < 0.001 < 0.001 = 0.172 = 0.001	− 0.323 (0.063) − 0.071 (0.066) − 0.581 (0.086) − 0.453 (0.056) − 0.163 (0.053) − 0.467 (0.070)	0.724 0.931 0.559 0.636 0.850 0.627	0.640–0.818 0.818–1.060 0.472–0.662 0.570–0.710 0.765–0.943 0.547–0.718	< 0.001 = 0.280 < 0.001 < 0.001 = 0.002 < 0.001
GCS ≤ 8 pre-hospital	− 0.455 (0.065)	0.634	0.559–0.720	< 0.001	− 0.687 (0.095)	0.503	0.417–0.606	< 0.001
SBP ≤ 90 mmHg	0.630 (0.032)	1.877	1.763–1.999	< 0.001	0.851 (0.045)	2.342	2.144–2.557	< 0.001
Age								
65–79 years ≥ 80 years	− 0.124 (0.029) − 0.334 (0.042)	0.883 0.716	0.835–0.934 0.660–0.777	< 0.001 < 0.001	0.295 (0.043) 0.275 (0.056)	1.344 1.316	1.236–1.461 1.179–1.470	< 0.001 < 0.001
ECS motor response								
Specific Unspecific No response	0.153 (0.033) 0.431 (0.088) 1.154 (0.069)	1.165 1.539 3.171	1.091–1.244 1.296–1.827 2.768–3.633	< 0.001 < 0.001 < 0.001	0.311 (0.052) 0.870 (0.127) 2.010 (0.098)	1.365 2.388 7.462	1.232–1.513 1.862–3.061 6.160–9.039	< 0.001 < 0.001 < 0.001

*ECS* Eppendorf–Cologne Scale

## Discussion

Our data describe a prevalence of 10.6% for relevant spinal trauma and 5.1% for severe cervical spine trauma in severely injured trauma patients in Europe. The prevalence of severe cervical spinal injuries described in our study is comparable to that found in studies of other countries [15, 17, 24]. Regarding potential predictors, independent variables were evaluated using regression analysis, which might yield differences compared to other calculations given the categories and characteristics used.

Blunt trauma was found to be predictive for a spinal injury and cervical spinal injury. Severe spine injury is four times more likely to result from a blunt trauma than a penetrating trauma. In line with the literature, this implies that patients with penetrating trauma should not be immobilized because spinal injury is unlikely and immobilization could delay lifesaving interventions [10, 25–28].

As expected, motor deficits in the clinical examination were an outstanding predictor for spine injuries, particularly cervical spine injuries. A lowered level of consciousness is often mentioned as a predictor of spinal trauma; however, in our regression analysis, a pre-hospital GCS < 9 was not a predictor for spinal injury. Our results corroborate those in the literature that, with severe isolated TBI or with TBI in combination with other injuries, severe injuries to the spine can be expected [29, 30].

Accident mechanisms are expected to provide an idea of the possible injuries, and discussions on this topic remain controversial [27, 31]. In our analysis, the documented accident mechanisms correlate poorly with cervical spinal injuries, although other studies have found otherwise [30]. Thus, we contend that an accident mechanism can only be considered as a risk factor that has to lead health professionals to an increased attention for cervical spinal injuries. However, falls from a height of more than 3 m were a precise predictor for an injury to the entire spine, which had already been confirmed by other studies [15, 27].

Patients aged 65 years and older were found to be more frequently affected by cervical spine injuries. The literature describes low-energy injuries, low-risk mechanisms, decreases in total mobility, and degenerative changes as causative factors [32, 33]. Additionally, a blood pressure ≤ 90 mmHg was found to be a significant predictor for the spine and cervical spine injuries. It was initially unclear whether hypotension is due to a neuronal disorder or hemorrhage. Table 1 shows that 41.3% of patients with a systolic blood pressure (SBP) ≤ 90 mmHg received blood transfusions in the shock room. Compared to the other characteristics, this is perhaps the most likely indication of hemorrhage in the context of overall injury severity.

That thoracic and abdominal injuries were most prevalent in the AIS 2–3 group confirms our expectations. This should be kept in mind for spinal injury, especially with increasing trauma severity and the number of accompanying injuries. Multiply injured patients in combination with TBI, as well as an expression of severely injured patients, are also more likely to have severe spinal cord injuries (AIS 4–6). Therefore, hemodynamic instability should be interpreted as a sign of high overall injury severity, which affects the spine in general and the cervical spine in particular. The fact that nearly 50% of the diagnostic certainty is faulty is regularly described in the literature and underlines the need for a simple but easy-to-use decision aid for (prehospital) immobilization [29, 34].

Currently, there are a mere handful of recommendations proposed for spinal immobilization [10, 11, 35]. These recommendations often consider the accident mechanism and abnormalities in the clinical examination equivalent, which failed to be confirmed by our analysis. Also, the German polytrauma guideline gives greater weight to clinical findings [27]. It is indisputable that life-threatening findings should take priority over immobilization measures [10, 11, 35].

Though pain is not documented in the TR-DGU, it would seem logical to take pain under consideration from a clinical standpoint. By way of example, a retrospective study has shown that immobilization was performed in 37.2% of patients due to back pain [36]. A cut-off for pain was not described, so that “the worse the pain, the more likely is immobilization” can be noted first, but immobilization is required, at the latest, when the patient requires analgesia. Healthcare professionals must be trained to interpret pain as a warning signal, but also to administer adequate analgesics [37]. Ultimately, however, as with every suspicion of benign fracture, a decision on whether splinting or immobilization is most appropriate must be made.

In case of an inconspicuous clinical examination and the presence of risk factors, immobilization does not seem to be superior to independent, pain-free relieving posture, although this does not preclude spinal injury [38–41]. Also, data show that the physical examination for possible spinal cord injury is not affected by the injury mechanism [31]. This raises the question of whether, due to the disadvantages of immobilization (e.g., discomfort, pressure points, restrictions of ventilation), “spinal motion restriction” exclusively is considered sufficient to prevent large, uncontrolled, passive movements (e.g., by transport) without securing the patient to a mattress or board.

For immobilization in TBI, it is important to know, that there are studies with minimal numbers of cases, some of which are almost 20 years old, which describe an increase in intracranial pressure (ICP) or increased internal jugular vein cross-sectional area when using a cervical collar [42–48]. A current meta-analysis with only 86 patients showed a significant overall increase in ICP after collar application (weighted mean difference = 4.43; 95% CI 1.70–7.17; *p* < 0.01) [49]. In this respect, the use of a cervical collar should certainly be avoided, and the cervical spine should be immobilized by other measures (manual, head blocks, etc.).

The much-described upper-body elevation (or head-of-bed elevation) in TBI has only weak evidence. In a Cochrane review on head-of-bed elevation in clinical care, no clear recommendation could be given [50]. In this respect, it must be considered for the acute phase whether an upper-body elevation, e.g., if isolated TBI is sensible, or whether additional injuries, physiological parameters of the rescue-specific aspects speak against it.

The best form of immobilization for transport (e.g., vacuum mattress, spineboard, stretcher) remains unclear despite numerous qualitatively heterogeneous, well-performed, and clinically relevant studies. How a patient must be immobilized or transported with low mobility remains a topic of active discussion. Some studies, for example, prefer the vacuum mattress for immobilization [51, 52]. Aside from the comfort of lying down, a recent exploratory biomechanical study found that the spineboard, followed by the stretcher with head immobilization, immobilizes better than the vacuum mattress [3]. Uzun et al. show that the remaining movement of the cervical spine is minimal when the patient is immobilized on a spine board with a headlock system or vacuum mattress with additional head blocks. The remaining movement of the cervical spine could not be reduced by the additional use of a cervical collar.[53]. So it seems to be superfluous. But it should be noted, however, that some paramedics—especially emergency physicians—lack proper immobilization skills [54]. Regular skills training, as well as risk assessment skills, are required to achieve improvements [55, 56].

It appears essential to keep in mind that immobilization does not influence neurological trauma; rather, it is merely intended to prevent further damage throughout the extrication and transport processes [6, 41, 57]. In addition to the clinical symptoms of pain, we see **s**pill (fall > 3 m), **s**eniority (age > 65 years), **s**eriously injured, and **s**kull/traumatic brain injury, which we term the “4S”, as an indication for increased attention by health professionals for cervical spinal injuries or indication for spinal motion restriction. The “4S” could prove useful as a memory aid, similar to the “3S” (scene, safety, situation) from emergency training for assessing a site.

As with all retrospective analyses, the present study has limitations. The trauma register does not include patients who died at the site of the accident; it only includes patients who reached the hospital alive. Given that we aimed to analyze severely injured persons, the applicability of our conclusions to patients who were less severely injured is questionable. We decided to include spine injuries from AIS ≥ 3 (serious injury = unstable fractures and neurological deficits). Stable fractures (AIS 2) are often diagnosed late and may, therefore, be less important for acute care during the initial stages of treatment. However, the AIS score estimates the severity of the injury rather than the impact of energy input, and the accident mechanism can only be differentiated rudimentarily because detailed information in the TR-DGU is not recorded.

## Conclusion

In summary, the results of this registry analysis should be included in the assessment of severely injured patients for the indication of spinal immobilization. For clinical evaluation, symptoms are more significant than kinematics. The risk factors (4S: spills, seniority, seriously injured, skull trauma) should focus the examination on the spine. If an examination is not possible because of impaired vigilance, the spine should be immobilized in case of doubt if the trauma mechanism is relevant. For logical considerations, in case of a vital risk, immobilization should not delay therapy or transport. Spinal injuries from isolated penetrating trauma should not be immobilized.

## Data Availability

Not applicable.

## Abbreviations

AIS: Abbreviated Injury Scale
CSI: Cervical spine injury
ECS: Eppendorf–Cologne Scale
ISS: Injury severity score
SCI: Spinal cord injuries
TBI: Traumatic brain injury
