# Correction to: Epidemiology and predictors of traumatic spine injury in severely injured patients: implications for emergency procedures

**DOI:** 10.1007/s00068-022-01914-1

**Published:** 2022-03-27

**Authors:** David Häske, Rolf Lefering, Jan-Philipp Stock, Michael Kreinest, The TraumaRegister DGU

**Affiliations:** 1 German Red Cross, Emergency Medical Service, Obere Wässere 1, 72764 Reutlingen, Germany; 2 Center for Public Health and Health Services Research, University Hospital Tübingen, Tübingen, Germany; 3 Institute for Research in Operative Medicine (IFOM), University Witten/Herdecke, Cologne, Germany; 4 Department of Anesthesiology, Intensive Care Medicine, Emergency and Pain Medicine, Klinikum am Steinenberg, Reutlingen, Germany; 5 Department of Trauma and Orthopedic Surgery, BG Trauma Center Ludwigshafen, Ludwigshafen, Germany; 6 Committee on Emergency Medicine, Intensive Care and Trauma Management (Sektion NIS) of the German Trauma Society (DGU), Cologne, Germany

## Correction to: European Journal of Trauma and Emergency Surgery 10.1007/s00068-020-01515-w

The original version of this article unfortunately contained a mistake. The presentation of Table 2 was incorrect. The corrected Table 2 is given below.

**Table 2:** Multivariate logistic regression analysis to predict any relevant spine injury (AIS 3–6, *n* = 15,481; prevalence = 10.6%), and relevant cervical spine injury (AIS 3–6, *n* = 5929; prevalence = 5.1%)

Predictor	Complete spine				Cervical spine			
	Coefficient (SE)	OR	95% CI	*p* value	Coefficient (SE)	OR	95% CI	*p* value
Head AIS ≥ 3	− 1.655 (0.032)	0.191	0.180–0.203	< 0.001	− 1.700 (0.047)	0.183	0.166–0.200	< 0.001
Face AIS ≥ 3	− 0.307 (0.039)	0.736	0.681–0.795	< 0.001	− 0.133 (0.056)	0.876	0.784–0.978	= 0.018
Thorax AIS ≥ 3	− 0.996 (0.024)	0.369	0.352–0.387	< 0.001	− 1.418 (0.040)	0.242	0.224–0.262	< 0.001
Abdomen AIS ≥ 3	− 0.838 (0.043)	0.432	0.397–0.471	< 0.001	− 1.131 (0.077)	0.323	0.277–0.375	< 0.001
Extremities and pelvis AIS ≥ 3	− 1.421 (0.032)	0.241	0.227–0.257	< 0.001	− 1.679 (0.056)	0.186	0.167–0.208	< 0.001
Male	0.009 (0.025)	1.009	0.961–1.059	= 0.730	0.210 (0.039)	1.234	1.144–1.332	< 0.001
Blunt trauma	1.403 (0.092)	4.066	3.395–4.870	< 0.001	1.292 (0.143)	3.640	2.748–4.821	< 0.001
Accident mechanism (reference car)								
Motorcycle Bicycle Pedestrian Fall > 3 m Fall < 3 m Other	− 0.007 (0.038) − 0.138 (0.048) − 0.534 (0.061) 0.808 (0.031) − 0.050 (0.037) − 0.152 (0.045)	0.993 0.871 0.586 2.243 0.951 0.859	0.922–1.070 0.793–0.956 0.520–0.660 2.109–2.385 0.885–1.022 0.787–0.939	= 0.852 = 0.004 < 0.001 < 0.001 = 0.172 = 0.001	− 0.323 (0.063) − 0.071 (0.066) − 0.581 (0.086) − 0.453 (0.056) − 0.163 (0.053) − 0.467 (0.070)	0.724 0.931 0.559 0.636 0.850 0.627	0.640–0.818 0.818–1.060 0.472–0.662 0.570–0.710 0.765–0.943 0.547–0.718	< 0.001 = 0.280 < 0.001 < 0.001 = 0.002 < 0.001
GCS ≤ 8 pre-hospital	− 0.455 (0.065)	0.634	0.559–0.720	< 0.001	− 0.687 (0.095)	0.503	0.417–0.606	< 0.001
SBP ≤ 90 mmHg	0.630 (0.032)	1.877	1.763–1.999	< 0.001	0.851 (0.045)	2.342	2.144–2.557	< 0.001
Age								
65–79 years ≥ 80 years	− 0.124 (0.029) − 0.334 (0.042)	0.883 0.716	0.835–0.934 0.660–0.777	< 0.001 < 0.001	0.295 (0.043) 0.275 (0.056)	1.344 1.316	1.236–1.461 1.179–1.470	< 0.001 < 0.001
ECS motor response								
Specific Unspecific No response	0.153 (0.033) 0.431 (0.088) 1.154 (0.069)	1.165 1.539 3.171	1.091–1.244 1.296–1.827 2.768–3.633	< 0.001 < 0.001 < 0.001	0.311 (0.052) 0.870 (0.127) 2.010 (0.098)	1.365 2.388 7.462	1.232–1.513 1.862–3.061 6.160–9.039	< 0.001 < 0.001 < 0.001

*ECS* Eppendorf–Cologne Scale

The original article has been corrected.

